# Quantitative metagenomics for marine prokaryotes and photosynthetic eukaryotes

**DOI:** 10.1093/ismeco/ycaf131

**Published:** 2025-07-30

**Authors:** Qicheng Bei, Nathan L R Williams, Laura E Furtado, Daria Di Blasi, Jelani Williams, Vanda Brotas, Glen Tarran, Andrew P Rees, Chris Bowler, Jed A Fuhrman

**Affiliations:** Department of Biological Sciences, University of Southern California, Los Angeles, CA 90089, United States; Department of Biological Sciences, University of Southern California, Los Angeles, CA 90089, United States; Department of Biological Sciences, University of Southern California, Los Angeles, CA 90089, United States; Department of Biological Sciences, University of Southern California, Los Angeles, CA 90089, United States; Department of Biological Sciences, University of Southern California, Los Angeles, CA 90089, United States; Faculdade de Ciências, Marine and Environmental Science Centre (MARE)/Aquatic Research Network (ARNET), Universidade de Lisboa, 1749-016, Lisbon, Portugal; Plymouth Marine Laboratory, The Hoe, Plymouth, PL1 3DH, United Kingdom; Plymouth Marine Laboratory, The Hoe, Plymouth, PL1 3DH, United Kingdom; Plymouth Marine Laboratory, The Hoe, Plymouth, PL1 3DH, United Kingdom; Institut de Biologie de l'École Normale Supérieure (IBENS), École Normale Supérieure, CNRS, INSERM, PSL Université, 75005 Paris, France; Research Federation for the Study of Global Ocean Systems Ecology and Evolution, FR2022/Tara GOSEE, 75016 Paris, France; Department of Biological Sciences, University of Southern California, Los Angeles, CA 90089, United States

**Keywords:** absolute quantification, metagenomics, internal standards, phytoplankton

## Abstract

High-throughput sequencing has provided unprecedented insights into microbial biodiversity in marine and other ecosystems. However, most sequencing-based studies report only relative (compositional) rather than absolute abundance, limiting their application in ecological modeling and biogeochemical analyses. Here, we present a metagenomic protocol incorporating genomic internal standards to quantify the absolute abundances of prokaryotes and eukaryotic phytoplankton, which together form the base of the marine food web, in unfractionated seawater. We applied this method to surface waters collected across 50°N to 40°S during the 29^th^ Atlantic Meridional Transect. Using the single-copy *recA* gene, we estimated an average bacterial abundance of 1.0 × 10^9^ haploid genome equivalents per liter. Leveraging a recent report that the *psbO* gene is typically single-copy in phytoplankton, we also quantified eukaryotic phytoplankton. Metagenomic estimates closely aligned with flow cytometry data for cyanobacteria (slope = 1.03, Pearson’s *r* = 0.89) and eukaryotic phytoplankton (slope = 0.72, Pearson’s *r* = 0.84). Compared to flow cytometry, taxonomic resolution for nano- and picoeukaryotes was greatly improved. Estimates for diatoms, dinoflagellates, and *Trichodesmium* were considerably higher than microscopy counts, likely reflecting microscopy undercounts and potential ploidy variation. These findings highlight the value of absolute quantification by metagenomics and offer a robust framework for quantitative assessments in microbial oceanography.

## Introduction

Absolute quantification of microorganisms has long been a critical aspect of microbiology for many decades, and as all aspects of environmental and biomedical microbiology have moved more and more towards molecular sequencing approaches, the need for absolute quantitation has not abated. It should be clear that in microbial ecological and biological oceanographic studies, absolute quantification of microorganisms is particularly advantageous, e.g. in ecosystem modeling and studies of microbially-mediated biogeochemical processes. Of particular interest are marine phytoplankton, which form the basis of the marine food web and whose identities and abundances set the tone for the entire ecosystem [1, 2].

Microbial 16S/18S ribosomal RNA (rRNA) gene sequencing has revolutionized environmental microbiology by advancing our understanding of microbial diversity and community structure. However, a key limitation of this approach is that it provides only relative abundance data, making it difficult to infer absolute cell numbers across samples [3]. To address this limitation, a known amount of synthetic or genomic DNA, referred to as “internal standard” or “spike-in”, can be added to samples prior to sequencing [4]. By relating read counts to these internal standards (ISDs), it becomes possible to estimate the absolute abundance of genes and, potentially, cells, assuming that gene copy number (GCN) per genome and ploidy are known. However, rRNA GCN remains unknown for a large fraction of naturally occurring prokaryotes [5] and eukaryotes [6], and PCR artefacts may further confound interpretation. These uncertainties complicate the accurate estimation of absolute cell abundances from amplicon-based sequencing data even when ISDs are used [7].

Shotgun metagenomics offers a promising alternative for estimating absolute microbial abundances by leveraging single-copy genes in environmental DNA. These genes, which, as the name implies, are typically present as one copy per genome, have been widely used in phylogenetic analysis [8, 9]. For example, the *recA* gene, which is highly conserved among bacteria species, involved in DNA repair, and readily recognizable informatically, is often used for taxonomic annotation and functional gene normalization [10, 11]. Since it generally occurs as a single copy per genome, *recA*-based metagenomics provides a way to estimate bacterial identities and cell counts (actually genome equivalents, because most bacteria are thought to be haploid). The archaeal homolog *radA* gene serves the same purpose [12]. Despite the concept of using ISDs in metagenomics is straightforward and elegant, it has only been applied in a limited number of marine studies to date. Notably, Gifford et al. used this approach to quantify prokaryotes in the eastern equatorial Pacific during an El Niño event [13], while Sharpe et al. showed a strong correlation between the *recA*-based estimates of *Synechococcus* and flow cytometry (FCM) data [14]. These studies demonstrated how this approach can be used for prokaryotes in marine environments.

While obtaining absolute abundances of prokaryotes metagenomically is extremely valuable, community and ecosystem-level ecological and biogeochemical studies require information on eukaryotes as well. Of special interest are marine phytoplankton, which contribute about half of the Earth’s primary production and oxygen generation [15]. Globally, marine phytoplankton production is roughly split between cyanobacteria and a broad variety of protists [16]. Traditionally, 18S rRNA gene sequencing has been the primary tool for profiling the community composition of protists. However, when trying to extrapolate to cell abundances (even relative ones) a major complication is that 18S rRNA gene copy number vary enormously, ranging from 1 to over 500 000 among known protists [6], especially high in dinoflagellates [17]. Growth phases and physiological states can also influence GCN [18]. To date, FCM remains an important method for rapidly counting smaller phytoplankton (“pico” and “nano” size ranges, up to ~20 μm diameter), whereas larger taxa classically require more laborious microscopy analyses or advanced imaging platforms such as the Imaging FlowCytoBot [19]. Only very recently has a “single-copy” gene been characterized for phytoplankton. Leveraging *Tara* Oceans data, Pierella Karlusich et al. pointed to the *psbO* gene [20], which encodes a subunit of the photosynthetic apparatus found in both cyanobacteria and eukaryotic phytoplankton. The *psbO* gene generally lacks non-photosynthetic homologs and is typically present as a single copy per haploid genome. However, many taxa, particularly diatoms and dinoflagellates, may exhibit diploid or polyploid states, with ploidy levels that can vary depending on life stage or environmental conditions [21]. The original paper showed its utility for estimating the *relative* abundance of taxa within whole phytoplankton [20]. However, this groundbreaking paper could only report relative abundances within size fractions, due to the absence of ISDs in the *Tara* Oceans multi-omics datasets.

Taking advantage of a new sampling opportunity, our goal here was to simultaneously quantify the absolute abundances of both prokaryotes (via *radA*, *recA*, and *psbO* for cyanobacteria) and eukaryotic phytoplankton (via *psbO*) in seawaters in units of haploid genome equivalents per liter. Samples were collected from unfractionated near-surface seawater during the 29^th^ Atlantic Meridional Transect (AMT29) research cruise. The AMT cruises cover a similar transect year after year, traversing from near the United Kingdom to waters off Argentina, and pass through multiple oceanic provinces with contrasting environmental conditions and biology (https://amt-uk.org/). To our knowledge, this is the first study to use metagenomics with ISDs to quantify both prokaryote and eukaryotic phytoplankton as genome equivalents per liter in whole, unfractionated seawater. Our approach provides a robust framework for future quantitative metagenomics in aquatic ecology.

## Materials and methods

### Sample collection

**Figure 1:**
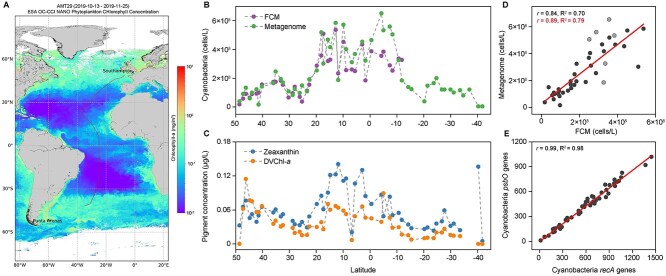
Broad agreement between metagenomics-based estimates of cyanobacterial absolute abundances and those made by FCM, as well as pigment patterns. (A) The AMT29 cruise track across the Atlantic Ocean is shown (see sampling stations in Fig. S1). The background color map represents the mean surface chlorophyll *a* concentration for October–November 2019 [22]. (B) Cell counts of cyanobacteria estimated using spike-in metagenomics and flow cytometry (FCM) methods. (C) Concentrations of cyanobacterial pigments zeaxanthin and divinyl chlorophyll *a* (DVChl-*a*) measured in surface samples. (D) Scatterplot between cyanobacterial abundances estimated by *recA*-metagenomics and FCM. Pearson’s correlations are presented for all samples (slope = 1.10) and separately for the first 33 stations (slope = 1.03) (darker dots only, red correlation statistics). (E) Scatterplot of cyanobacterial haploid genome equivalents based on *recA* and *psbO* (slope = 0.72; Pearson’s *r* = 0.99) gene markers through spike-in metagenomics.

Samples were collected during the AMT29 cruise aboard the RRS Discovery, which sailed from Southampton (UK) to Punta Arenas (Chile) from October 13 to November 25, 2019 (Fig. 1). The ship stopped twice daily (around 4 a.m. and 12:30 p.m. local time) along the transect, and sampling was conducted from surface waters (2–5 m) using a rosette equipped with 24 Niskin bottles (Fig. 1A). At each station, ~1 L of seawater was collected from a Niskin bottle and pumped through a 0.22 μm Sterivex filter (PVDF, Millipore, SVGVL10RC). After filtering, 0.5 ml RNA Later Solution (Invitrogen by Thermo Fisher Scientific) was added to the Sterivex filter for sample preservation, and filters were stored at −80°C until extraction. The temperature, salinity and chlorophyll-*a* fluorescence (Chl*a*) in the upper 200 m were measured using a conductivity-temperature–pressure probe (SeaBird, SBE, 911plus/917) (Cruise report of AMT29, https://dx.doi.org/10.17031/t8ed-w534). In addition, phytoplankton pigment concentrations along the transect were analyzed using High Performance Liquid Chromatography (HPLC) [22].

### DNA extraction and internal standards

First, RNAlater was removed from the Sterivex filter by brief centrifugation with inlet side down. The Sterivex filter was then rinsed with TE buffer, and any suspended DNA was desalted and recovered from the RNAlater plus TE rinse by centrifugal ultrafiltration (Centricon, three cycles). Preliminary studies showed this recovery was necessary because we often found DNA in the RNAlater. This desalted nucleic acid fraction was added back to the crude extract for further purification. Sterivex filters were aseptically opened using sterile pliers in petri dishes. The filter was removed from plastic housing, cut into small strips using sterile blades and forceps, and added to bead beating tubes along with the liquid nucleic acid fraction and RLT lysis buffer from the AllPrep DNA/RNA mini kit (Qiagen, Valencia, CA, USA).

Cells were lysed using bead beating with 0.1–0.5 mm zircon beads, followed by total nucleic acid purification with the AllPrep DNA/RNA mini kit (Qiagen, Valencia, CA, USA). For quantitative analysis, three genomic standards (*Thermus thermophilus* ATCC BAA-163, *Blautia producta* ATCC27340, *Deinococcus radiodurans* ATCC13939) were added to the lysis buffer after bead beating (crude DNA extraction), targeting ~1% of total DNA content as internal standards [13]. DNA extraction and purification generally followed manufacturer instructions, with full details available at https://www.protocols.io/workspaces/fuhrman-lab.

### Metagenome sequencing and absolute quantification

A total of 53 AMT29 samples were spiked with ISDs and sequenced. DNA libraries were prepared and barcoded using the NEBNext® Ultra™ II FS DNA Library Prep Kit (New England Biolabs). The metagenomic libraries were sequenced on an Illumina NovaSeq platform at the Tufts University Core Facility (Boston, MA, USA) using 2 × 250 bp paired-end sequencing. When we later recognized during the study that we desired deeper coverage to obtain more *psbO* hits than in the original sequencing outputs, 10 samples from AMT29 were re-sequenced using an AVITI sequencer (2 × 300 bp mode) at the University of Minnesota Genomics Facility (UMGC).

For metagenomic estimation of absolute gene abundances, we followed the approach outlined by Gifford et al. [13]. Briefly, raw reads were quality-trimmed using Trimmomatic v0.39 [23]. Paired-end reads were assembled using PEAR v0.9.6 [24]. Reads from the internal genomic standards were identified via BLASTn (e-value <0.001, %id >95%, alignment length 50%, bit score > 50), followed by BLASTx searches (e-value <0.001, %id >98%, bit score > 50). Bacterial *recA* and archaeal *radA* proteins were downloaded from the RefSeq protein database (2024.11), and metagenome reads were compared to the databases using DIAMOND v2.1.9 [25] (BLASTx, −e 0.001, −k 1, %id >80%, bit score > 50) [13]. Proteins sequences were also verified with GhostKOALA against the Kyoto Encyclopedia of Genes and Genomes webserver [26]. The taxonomies of *recA* and *radA* genes were determined by aligning the sequences to the NCBI nr database (2024.11) using DIAMOND (BLASTx, −e 1e-5, −k 20), and the outputs were summarized using MEGAN v7 community edition [27] with the GTDB taxonomy. For the *psbO* gene, assembled reads were searched against the database generated from *Tara* Oceans datasets using BLASTn (e-value <0.001, %id >80%, bit score > 50) [20].

Recovery of internal standards in the metagenomics was used to estimate gene volumetric abundances for each sample using calculations partly derived from Gifford et al. [13]:

(1) *S*_r_ = $\frac{S_{\mathrm{S}}}{S_{\mathrm{P}}}$ (2) *R* = $\frac{S_{\mathrm{r}}}{S_{\mathrm{a}}}$ (3) *G*_a_ = $\frac{G_{\mathrm{s}}}{R}$ (4) *G*_euk_ = *G*_recA_cyano_  $\times \frac{PsbO_{\mathrm{euk}}}{PsbO_{\mathrm{cyano}.}}$

(1) *S*_r_: Copies of internal standard genome recovered from sequencing in the sample.

*S*_S_: all reads matching protein-coding internal standard genes in the sample (BLASTx against genes used for *S*_p_).

*S*_p_: all protein-coding genes in the internal standard reference genome.

(2) *R*: Recovery ratio. The proportion of added standard molecules that were recovered through sequencing in the sample.

*S*_a_: Copies of internal standard genome added to the sample.

(3) *G*_a_: Molecules of a given gene (e.g. *recA* gene from a particular organism).

*G*_s_: total reads of the same gene (as used for *G*_a_) in the sample.

(4) *G*_euk_: Haploid genomes of a particular photosynthetic eukaryote (or aggregated into broader groups) in the sample.

*G*_recA_cyano_: Total cyanobacterial *recA* reads in the sample (i.e. *G*_a_ where a is *recA* from all cyanobacteria).

*PsbO_euk_*: Reads of a particular eukaryotic *psbO* in the sample (same identity as *G*_euk_).

*PsbO_cyano_*: Total cyanobacterial reads identified as *psbO* in the sample.

The volumetric abundance of each single-copy gene (e.g. *radA* or *recA*) was determined by dividing gene counts by the volume of seawater filtered. The *psbO* gene is encoded by both cyanobacteria and eukaryotic phytoplankton, enabling cross-domain analysis. We employed two approaches to estimate haploid genome equivalent of photosynthetic eukaryotes. The first applied the recovery ratio directly to *psbO* reads, as done for *recA* (Equation 3). However, this method underestimated cyanobacterial (*Prochlorococcus* and *Synechococcus*) abundances, with a slope of 0.72 (Pearson’s *r* = 0.99) compared to *recA*-based estimates (Fig. 1). As an alternative, we calculated the ratio of taxon-specific eukaryotic *psbO* reads to cyanobacterial *psbO* reads and then multiplied it by the absolute cyanobacterial abundance based on *recA* (Equation 4). This assumes an accurate *recA* cyanobacteria count and that *psbO*-based underestimation similarly affects both cyanobacteria and photosynthetic eukaryotes, likely due to sequencing depth and limitations in the *psbO* reference database (see Discussion).

### Amplicon sequencing and data analysis

DNA samples from AMT29 with internal standards were sequenced for amplicons targeting the V4-V5 hypervariable region of the 16S and 18S rRNA gene using the universal primers 515Y/926R [28], as described in the protocol at doi.org/10.17504/protocols.io.vb7e2rn. The primers amplify prokaryotes, eukaryotes, and chloroplasts simultaneously. The amplicon library was sequenced using an AVITI sequencer at UMGC using 2 × 300 bp mode. The sequences were demultiplexed and denoised to amplicon sequence variants based on DADA2 [29] incorporated within QIIME2 [30] with a special protocol that captures 18S sequences, all of whose forward and reverse reads do not overlap and are lost with standard protocols [31].

### Cell counting by microscopy and flow cytometer

Microscopy was used to identify cells with an equivalent spherical diameter (ESD) ≥ 10 μm, while the flow cytometer targeted smaller cells with ESD < 10 μm (Table S1). For each sampling site, 200 ml samples were collected in amber glass bottles and fixed with neutral Lugol’s iodine solution. In the laboratory, cell identification and counting were performed using a Zeiss Axiovert 200 inverted microscope with 10 × 40 magnification. Major eukaryotic species from diatoms, autotrophic and heterotrophic dinoflagellates were identified and counted. For *Trichodesmium* (filamentous Cyanobacteria), cell counts were estimated for each filament, giving an average number of 100 cells per filament [22]. Fresh samples were also analyzed for smaller-celled phytoplankton abundances using a Becton Dickinson FACSort flow cytometer. Within the analysis window, six different groups were enumerated: *Prochlorococcus, Synechococcus*, picoeukaryotes (PEUK), nanoeukaryotes (NEUK), coccolithophores within 5–10 μm, and cryptophytes, as previously reported [22].

## Results

### Quantitative metagenomics compared to FCM and pigments

The AMT29 cruise spanned >6200 km the northern and southern Atlantic between the United Kingdom and Argentina (Fig. 1A, Fig. S1). Lowest Chl*a* concentrations occurred in oligotrophic gyres, moderate levels in the tropics (especially the northern tropics, closest to Africa on this transect), and highest values at higher latitudes. Cyanobacterial absolute abundance, inferred from the *recA* sequences and recovery ratio of ISDs (Fig. S2, Tables S2), exhibited a 140-fold variation across stations, peaking at 6.7 × 10^8^ cells L^−1^ in the tropics (Fig. 1B). Similarly, the pigment biomarker zeaxanthin, indicative of *Synechococcus* and *Prochlorococcus*, peaked in the northern tropics (Fig. 1C). Divinyl chlorophyll-*a*, a marker for *Prochlorococcus*, followed the same trend (Fig. 1D).

A strong linear correlation with a slope near 1 was found between cyanobacterial cell counts derived from *recA*-based metagenomics and FCM, most strongly for the first 33 stations (slope = 1.03; Pearson’s *r* = 0.89) (Fig. 1D, Table S3); we calculated separately for these 33 because the FCM data was unusable for *Prochlorococcus* between 12°S and 40°S, and data from six stations closest to those show worse correlation and may be suspect. Cyanobacterial *psbO* and *recA* genes correlated strongly (slope = 0.72; Pearson’s *r* = 0.99), though the slope indicates an average of 28% fewer *psbO* hits detected compared to *recA* (Fig. 1E, Fig. S3), possibly due to some *psbO* fragments not being clearly recognized as such informatically.

### Relative and absolute profiling of prokaryotes along the AMT29 transect

**Figure 2:**
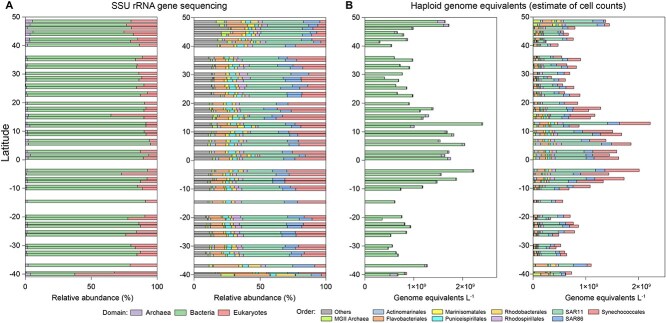
Relative and absolute abundance estimates in AMT29 samples. (A) Relative abundance of major taxa at the domain (left) and order (right) levels derived from universal 3-domain rRNA amplicon sequencing. (B) Absolute abundance of major prokaryotic taxa at the domain (left) and order (right) levels estimated using quantitative metagenomics via *recA* for bacteria and *radA* for archaea.

SSU rRNA gene sequencing using three-domain universal primers (515Y/926R), revealed average relative rRNA gene abundance of archaea (1.8%), bacteria (82.8%), and eukaryotes (15.4%) across the AMT29 transect (Fig. 2A). The most abundant prokaryotic taxa included SAR11, *Synechococcales*, *Flavobacteriales*, *Pseudomonadales*, *Puniceispirillales*, and *Rhodobacterales*, with marine group II (MGII) dominating the archaeal community (Fig. 2A).

Quantitative metagenomics estimated bacterial cell counts (genome equivalents from *recA*) ranged 3.0 × 10^8^ to 2.5 × 10^9^ cells L^−1^, while archaeal counts (from *radA*) varied between 5.7 × 10^5^ and 1.5 × 10^8^ cells L^−1^ (Fig. 2B, Table S4). Prokaryote abundance broadly paralleled the changes in Chl*a* concentrations (Fig. 2B); lowest abundances were observed in both Southern and Northern oligotrophic gyres, and highest in the tropics, which on this transect had moderate chlorophyll levels (Fig. 1A). Abundance patterns of major taxa were strongly consistent between metagenomic and rRNA amplicon-based microbial profiling (Fig. 2), though some differences were evident when the metagenomic data were viewed as relative abundance; this may be expected due to variable rRNA GCN and possible DNA extraction and PCR biases (Fig. S4A). The highest correlation coefficient between the relative abundances by the two measures was observed for *Synechococcales* (Pearson’s *r* = 0.95), while the lowest was for *Puniceispirillales* (SAR116) (Pearson’s *r* = 0.72) (Fig. S4B). The average cell counts for *Prochlorococcus* and *Synechococcus* were 2.3 × 10^8^ and 8.4 × 10^6^ cells L^−1^, respectively. *Prochlorococcus* dominated the cyanobacterial community from 46°N to 35°S, whereas *Synechococcus* peaked particularly strongly at the southernmost station (40°S) (Fig. S5A). Metagenomics resolved to the ecotype level, unlike FCM (Fig. S5B). Microbial community composition varied more distinctly when assessed using absolute abundance (68%) compared to relative abundance (53%), as shown by more of the variance explained in the PCoA of the Bray–Curtis dissimilarity matrix (Fig. S6).

### Quantifying eukaryotic phytoplankton along the AMT29 transect

**Figure 3:**
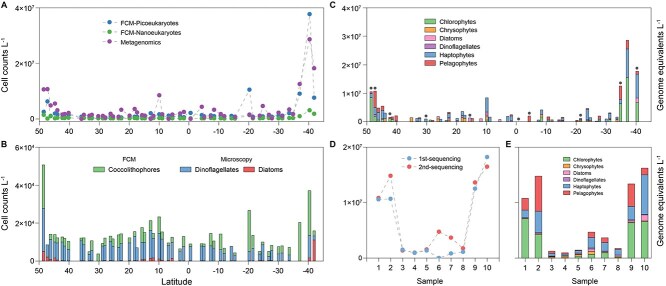
Comparison of abundances of eukaryotic phytoplankton show metagenomics broadly agrees with FCM of nano-and pico-eukaryotes, but metagenomics-based abundances of dinoflagellates and diatoms were much higher than microscopy. (A) Total eukaryotic haploid genome equivalents, estimate of cell counts via equation (4)—see text, calculated using *psbO*-based metagenomics, alongside nano- and pico-eukaryote counts determined by FCM. (B) Eukaryotic cell counts derived from FCM for coccolithophores and from microscopy for dinoflagellates and diatoms. (C) Cell counts of major eukaryotic groups identified using *psbO* genes, i.e. the same metagenome results as in panel A, broken down by major groups. Note the numerically dominant groups from metagenomes are expected to be largely in the pico and nano size range, with cell diameters <20 μm (see text). Samples marked with circular dot were resequenced. (D) Total eukaryotic cell count estimates in the 10 resequenced samples. The resequencing effort yielded 26 289 *psbO* sequences, comprising 25 079 from cyanobacteria and 1210 from eukaryotes. (E) Absolute abundance estimates from (D) divided into major of eukaryotic groups.

Diatoms and dinoflagellates were counted via microscopy, while coccolithophores, cryptophytes, NEUK, and PEUK were quantified by FCM (Table S1). Along the transect, PEUK (86.0%), and NEUK (13.2%) together accounted for over 99% of eukaryotic phytoplankton (Fig. 3A), with peak abundances near 40°S latitude. Dinoflagellate and diatom microscopy abundances peaked at stations around 48°N and 40°S, respectively (Fig. 3B). Coccolithophores peaked (2.4 × 10^4^ cells L^−1^) by FCM at 40°S (Fig. 3B, Table S1). Coccolithophores and dinoflagellates exhibited elevated abundances in the tropics as well as northern and southern extremes of the transect, and lowest in the subtropical gyres (Fig. 3B). Amplicon sequencing indicated that chlorophytes, dinoflagellates and haptophytes, along with Metazoa, tended to dominate the eukaryotic community by this measure, very likely affected by 18S rRNA gene copy number variations (Fig. S7).

Metagenomic analysis of the AMT29 dataset yielded 22 388 *psbO* sequences, comprising 22 077 from cyanobacteria and 311 from eukaryotes in the initial round of sequencing (Table S5) (note we re-sequenced 10 samples to increase eukaryote coverage, see below). We differentiated six eukaryotic phytoplankton groups: chlorophytes, chrysophytes, diatoms, dinoflagellates, haptophytes, and pelagophytes (Fig. 3C). The metagenomics-derived abundances of photosynthetic eukaryote (assuming one *psbO* gene per haploid genome) showed strong correlation with total cell counts by microscopy and FCM (slope = 0.72; Pearson’s r = 0.84) (Fig. S8). Chlorophytes and haptophytes dominated the phytoplankton community, with peaks near 40°S latitude. Among chlorophytes, *Bathycoccus*, *Micromonas*, and *Ostreococcus* were the most abundant genera (Table S6). Haptophytes included the coccolithophore *Emiliania* and non-coccolithophore genera *Chrysochromulina* and *Prymnesium*. The pelagophyte *Pelagomonas* also showed higher abundance at higher latitudes in both hemispheres. Diatoms and dinoflagellates were also detected, with cell counts exceeding 1 million cells L^−1^ from 15°N to 10°N (Fig. S9). A relatively high abundance of chrysophyte-like sequences (~70% BLAST identity) was observed from 20°N to 10°N. Resequencing of 10 samples yielded 13-fold higher coverage (Table S7), yet community composition and absolute abundance remained remarkably consistent between the two sequencing runs (Fig. 3D-E).

## Discussion

Metagenomic methods incorporating internal standards for absolute quantitation of prokaryotes were developed over a decade ago [32], and in recent years have been applied to estimate genome equivalent (or haploid equivalent) cell counts with the analysis of single-copy genes in some marine studies [11, 13]. Nevertheless, the vast majority of metagenomic studies still reported only relative abundances. Until this past year there was no recognized single-copy gene allowing for genome equivalent quantification of phytoplankton, and still no known general protistan single-copy gene to assess heterotrophic protist abundances. By integrating ISDs with single-copy *recA* and *psbO* genes, our study establishes a robust workflow for estimating cell counts, more specifically counts of haploid genome equivalents, across all three domains of life, and we applied it along the Atlantic Meridional Transect. The strength of this approach is supported by the strong correlations and similar absolute estimates observed between phytoplankton abundances derived from quantitative metagenomics and those obtained using FCM and microscopy measurements: for cyanobacteria, Pearson’s *r* = 0.89; for photosynthetic eukaryotes, Pearson’s *r* = 0.84.

Historically, microscopy, FCM and quantitative PCR (qPCR) have been the primary methods for estimating absolute cell abundance. While qPCR is highly sensitive and specific for microbial detection, its limitations include usually targeting only specific groups, variability in PCR efficiency, primer specificity, and the common presence of multiple gene copies per cell [33]. FCM, on the other hand, provides high-throughput cell counts but requires supervised denoising/differentiation and offers very limited taxonomic resolution [34]. In this study, spike-in metagenomics revealed cyanobacterial cell estimates highly correlated to and numerically equivalent to those obtained via FCM. Note that a recent paper reported virtually identical FCM and rRNA amplicon-based estimates of *Prochlorococcus* and *Synechococcus* counts in a North Pacific transect when these same kind of spike-ins were used and it was assumed that *Prochlorococcus* has one rRNA gene copy while *Synechococcus* has two [35]. In our study only a few moderate discrepancies were observed between FCM and *recA* gene analyses of cyanobacterial abundance, and it was in the tropics, where intense sunlight and low cellular pigment concentrations may have led to an underestimation of near-surface marine cyanobacteria by FCM [36]. So in addition to being very versatile, detecting all taxa with good phylogenetic resolution, quantitative metagenomics may help make up for the possible deficiencies in FCM.

In our study, prokaryotic community profiles showed broad quantitative agreement between amplicon sequencing and metagenomics in terms of relative abundance (Fig. S4). We find this encouraging in that it means that carefully planned SSU rRNA amplicon studies (e.g. vetted with mock communities and metagenomic comparisons as is the case for the primers and protocols used here [28, 37]) in marine plankton investigations like this yield relative abundances that reasonably reflect cell abundances. But we do recognize that variations in rRNA GCN among prokaryotes would need to be considered for specific comparisons [38], e.g. it is thought to be 1 in most *Prochlorococcus* and SAR11, 2 in most *Synechococcus*, 1–6 in *Rhodobacter*, and 1–12 in *Flavobacterium* etc. Our study also highlights the challenge of directly comparing prokaryotes and eukaryotes even when the relative abundances of rRNA genes are measured with a single denominator. Eukaryotes accounted for around 10% of all SSU rRNA amplicons, ~30-fold higher than the ~0.3% eukaryote proportion of the total haploid genome equivalents (~cells) from the metagenomic data. This almost certainly reflects the higher rRNA GCN in eukaryotes as reported in a previous study (up to hundreds) [39]. Providing protist GCN in field samples remains very challenging. So because ecologists and modelers are much more interested in cell abundances than gene copy numbers alone, “single-copy” gene quantification like the ones we report here have clear advantages.

By being an all-taxon approach, our single analysis was able to indicate cell abundances, as haploid genome equivalents, of all prokaryotic and phytoplankton taxa (that exceed detection limits), something new for large ocean transects. Among prokaryotes, members of the SAR11 clade, the most abundant plankton group in marine systems, were uniformly high throughout this surface transect, averaging 3.6 × 10^8^ cells L^−1^, accounting for approximately one-third of the prokaryotes. *Synechococcales* (*Prochlorococcus* and *Synechococcus*), the most abundant primary producers, averaged 2.4 × 10^8^ cells L^−1^, or 21.9%, particularly dominant in warm waters as expected. The MGII archaea averaged 2.9 × 10^7^ cells L^−1^, 2.6% of the total prokaryotes, and peaked in abundance at the highest latitudes. Marine diazotrophs, crucial for nitrogen cycling [40], were also quantified. Notably, we report the diazotroph *Trichodesmium* at eight stations exhibited an average abundance of 1.5 × 10^6^ genome equivalents L^−1^ between 1°N and 22°N (Fig. S9), a 325-fold increase compared to microscopy-based estimates [22] from the same cruise. Microscopy estimates for *Trichodesmium* were based on filaments, using an average of 100 cells per filament [22]. Previous studies also reported high *Trichodesmium* abundances in the Atlantic Ocean between 0°N and 15°N across AMT1–8, estimating an average of 300 ± 100 filaments per liter [41]. The much higher metagenomic estimates may reflect high abundance of small filaments or single cells that were missed microscopically. However, it could in part reflect that *Trichodesmium* may sometimes be polyploid, reportedly containing up to 100 genome copies per cell [42]. Another important marine diazotroph, the symbiotic unicellular cyanobacterium *Candidatus Atelocyanobacterium thalassa*, or “nitroplast” [43], was abundant (~1.3 × 10^6^ cells L^−1^) between 10°S and 35°S in the South Atlantic Ocean (Fig. S10), within the reported range from prior work [44]. Unlike microscopy and qPCR [44] methods, our approach provides direct quantification of marine diazotrophs along with every other organism (with no extra effort or cost), providing particularly valuable ecological context about the entire community at once.

Traditional identification of large marine phytoplankton, such as diatoms and dinoflagellates, relies on light microscopy, with highly sensitive detection thresholds as low as 20 cells L^−1^. However, this method is time-intensive and requires specialized expertise. With the decreasing costs of sequencing, metagenomic detection thresholds can be significantly improved. For example, our resequencing of 10 AMT29 samples greatly enhanced *psbO* gene recovery, reducing the average detection threshold from 0.58 million to 68 000 haploid genome equivalents L^−1^. Notably, metagenomics showed >1 million diatom and dinoflagellate haploid genome equivalents L^−1^ at stations between 10°N and 15°N, representing a ~100-fold increase compared to microscopy cell count estimates. This finding supports earlier studies suggesting that substantial numbers of nano-sized diatoms and dinoflagellates exist in some coastal and ocean areas [45, 46]. Due to the small size and detection challenges, they remain poorly characterized. Discrepancies may also stem from resistance of some organisms to cell lysis and DNA extraction (e.g. some archaea, cysts, or others with rigid cell walls) and preservation biases such as cell lysis in RNAlater, which we accounted for but may be overlooked in other protocols. Also note we used a fairly harsh bead beating step in our extractions to maximize lysis. Diatoms are generally thought to be diploid in their vegetative state, whereas most dinoflagellates follow haplontic life cycles [21], potentially leading to overestimation of phytoplankton abundances by *psbO* genes (i.e. for diploid organisms, the number of cells is half the haploid genome equivalents).

We also noted that for cyanobacteria, the annotated metagenomic *psbO* read counts were beautifully correlated but consistently only ~72% as high as *recA* based counts (Fig. 1E, regression slope 0.72), while *recA* counts of cyanobacteria closely matched flow cytometry and thus appeared accurate. We used this information to develop a second way of estimating eukaryotic phytoplankton haploid genome equivalents, beyond the one based solely on *psbO* absolute abundance (via Equation 3), to compensate somewhat for the likely *psbO* undercount. This alternative calculation (Equation 4) takes the ratio of taxon-specific eukaryotic *psbO* reads to total cyanobacterial *psbO* reads within a given sample and multiplies this by the total cyanobacterial *recA* genes in the sample, leveraging the accurate *recA* cyanobacterial estimates. It basically assumes the eukaryotic *psbO* undercount is the same as that observed for prokaryotes. We speculate the undercount is largely from the difficulty matching short reads to the currently sparse *psbO* database at high identity. Further improvements in the spike-in metagenomics, including increased sampling volume, deeper sequencing, expanded *psbO* gene reference databases, determination of ploidy levels and cell cycle stages, and possibly alternate lysis methods, are expected to enhance the accuracy of absolute quantification for eukaryotes [47].

Nano- and pico-eukaryotes play critical roles in marine ecosystem diversity and functionality. Over recent decades, microscopy and FCM studies have demonstrated their high abundances [48]. In the Atlantic Ocean, the latitudinal distributions of NEUK and PEUK were largely consistent between FCM and metagenomics, and in fact these organisms made up 99% of the total *psbO* sequences. Notably, quantitative metagenomics significantly improved taxonomic resolution, enabling the detection of orders such as *Isochrysidales* and *Prymnesiales* (nanoeukaryotes), and *Pelagomonadales* and *Mamiellales* (PEUK). The *psbO* sequences also showed uncultivated haptophytes (~80% identity) related to *Chrysochromulina*, *Emiliania* (*Gephyrocapsa*), and *Prymnesium*. Absolute abundances of pelagophytes, chlorophytes and haptophytes peaked in high-latitudes, where some are known to make massive blooms visible from space [49]. Haptophytes, including toxin-producing members of genera such as *Prymnesium* and *Chrysochromulina*, can cause harmful algal blooms [50], so determining absolute abundances of potentially toxic genera metagenomically (and examining the corresponding metagenomes more closely for indicators of potential toxicity) can help understand their ecology. Genera of green algae, such as *Bathycoccus*, *Micromonas*, and *Ostreococcus*, are important contributors to primary production and are abundant globally, including in Arctic waters [51, 52]. Thus, quantitative estimation of taxonomically identified pico- and nano-eukaryotes is particularly important in the context of understanding primary production and biogeochemical cycling, and how it may be influenced by global warming, especially in climate-sensitive polar regions [53].

Although photosynthetic eukaryotes accounted for an average of only 0.3% of the haploid genome equivalents, their individual cell biovolumes (and corresponding biomasses) can be several orders of magnitude larger than that of prokaryotes, so their potential contribution to biomass is significant. Spike-in metagenomics would facilitate the phytoplankton carbon biomass estimates when augmented with taxon-specific estimates of biomass per cell, which have been used for decades in classical microscopy methods [9]. However, this approach would require substantial additional effort, as well as some speculation at this time, so it is beyond the scope of this report.

In summary, quantitative metagenomics now provides a robust framework for estimating taxonomically resolved absolute cell counts by integrating ISDs with single-copy genes such as *recA* and *psbO* [13]. This dual-gene strategy enables simultaneous absolute quantification of plankton across domains by a single assay. Our study extends previous spike-in metagenomics by providing a quantitative assessment of prokaryotes and photosynthetic eukaryotes across the Atlantic Meridional Transect. Future applications can include taxonomically resolved estimates of carbon biomass by incorporating cell-specific biomass data, as well as absolute quantification of specific viruses. Combining satellite-derived global surface Chl*a* concentrations with taxonomically resolved metagenomics presents a promising approach to improve estimates of primary productivity on a global scale [54].

Author contributions: Q.C.B. conducted molecular experiments, performed bioinformatics, and drafted the manuscript. N.W. performed molecular measurements and carried out bioinformatics analyses of amplicons. L.F., D.D.B and J.W. contributed to the molecular experiments. V.B. provided microscopy and HPLC pigment analyses. G.T. conducted flow cytometry experiments. A.R. leads the AMT project, organized the cruise and assisted with sample collection. C.B. provided insights into databases and results interpretation. J.A.F. conceived the study and supervised the project. Q.C.B. and J.A.F. wrote and finalized the manuscript with input from all authors.

## Supplementary Material

Supplementary-Figures-ISME_Comm_ycaf131

Supplementary-Tables-ISME_Comm_ycaf131

## Data Availability

Raw sequence data of AMT29 have been deposited to the NCBI under accession: amplicon sequencing (PRJNA1226253) and metagenome with internal standards (PRJNA1194529 and PRJNA1194620). Databases of the single-copy genes (*radA* and *recA*) used for this study can be accessed through Figshare (https://doi.org/10.6084/m9.figshare.28921349.v1). The scripts used for metagenomics with internal genomic standards are available at github.com/beiqicheng/.
