# Supplementary material for: Quantitative metagenomics for marine prokaryotes and photosynthetic eukaryotes
Source: ISME Commun. 2025 Jul 30;5(1):ycaf131. doi: 10.1093/ismeco/ycaf131 (PMC12378644; doi:10.1093/ismeco/ycaf131)
Supplement: Supplementary-Figures-ISME_Comm_ycaf131 [file supplementary-figures-isme_comm_ycaf131.pdf]

Supplementary Information for

Quantitative metagenomics for marine prokaryotes and photosynthetic eukaryotes

Qicheng Bei[1, *], Nathan L. R. Williams[1], Laura E. Furtado[1], Daria Di Blasi[1], Jelani Williams[1], Vanda Brotas[2,3], Glen Tarran[3], Andrew P. Rees[3], Chris Bowler[4, 5], Jed A. Fuhrman[1, *]

[*]Corresponding authors:

Qicheng Bei: qbei@usc.edu

Jed Fuhrman: fuhrman@usc.edu

This PDF file includes:

Quantitative metagenomics pipeline

Figures S1 to S10

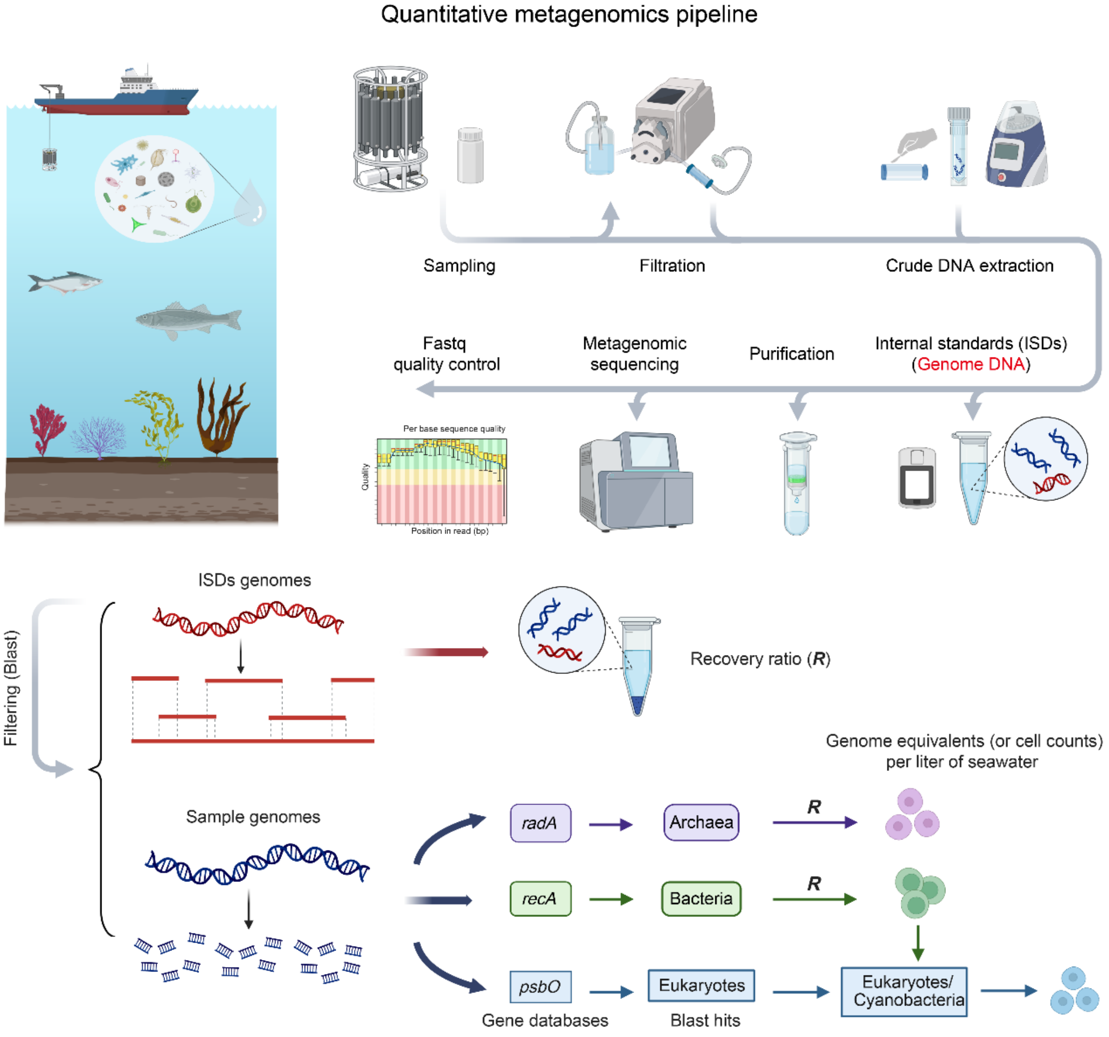
Quantitative metagenomics pipeline
Sampling
Filtration
Crude DNA extraction
Fastq
quality control
Metagenomic
sequencing
Purification
Internal standards (ISDs)
(Genome DNA)
Per base sequence quality
Quality
Position in read (bp)
ISDs genomes
Filtering (Blast)
Recovery ratio (R)
Genome equivalents (or cell counts)
per liter of seawater
Sample genomes
radA
Archaea
R
recA
Bacteria
R
psbO
Eukaryotes
Eukaryotes/
Cyanobacteria
Gene databases
Blast hits

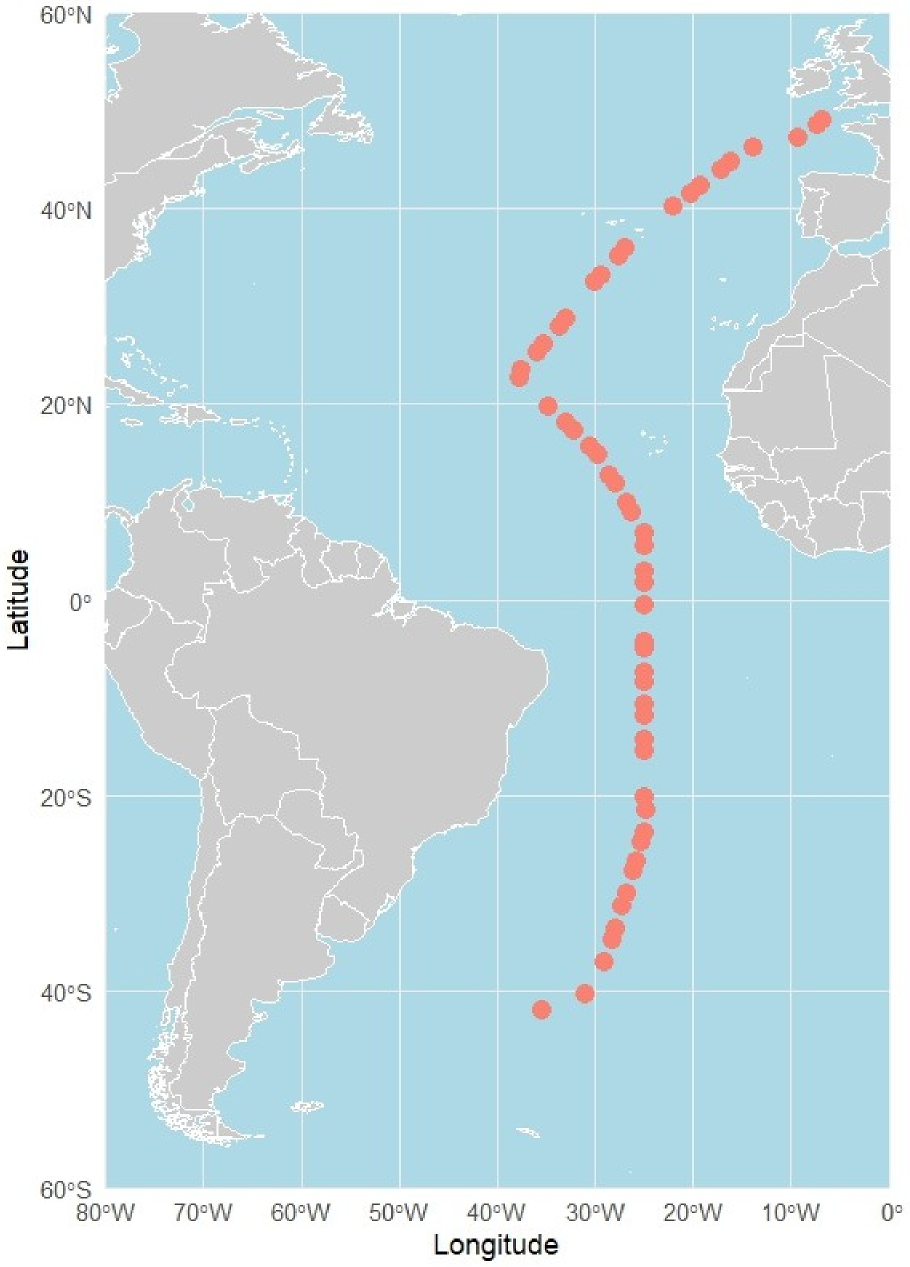

Fig. S1. Location of sampling stations on the AMT29 cruise.

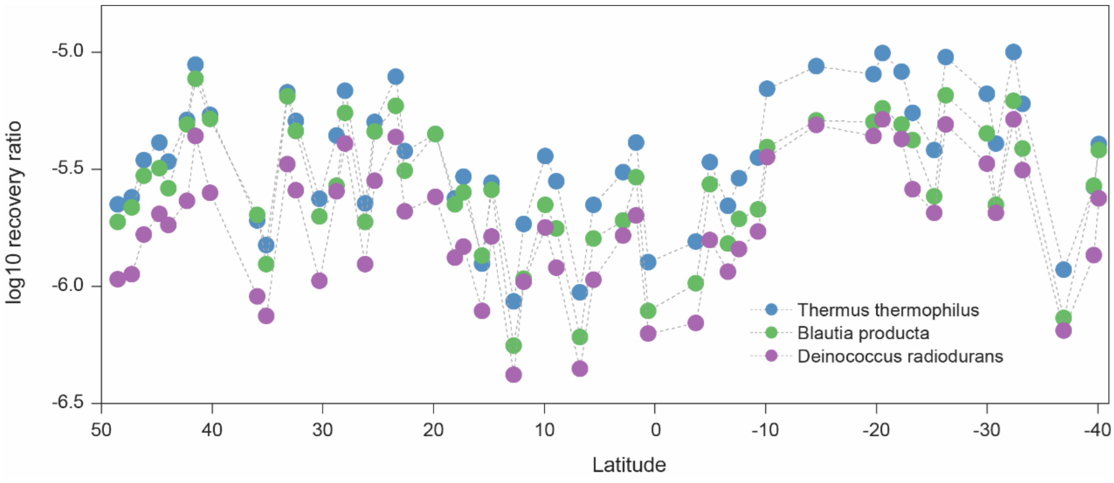

Fig. S2. Log10-scaled recovery ratios for the three internal standards in AMT29 samples.

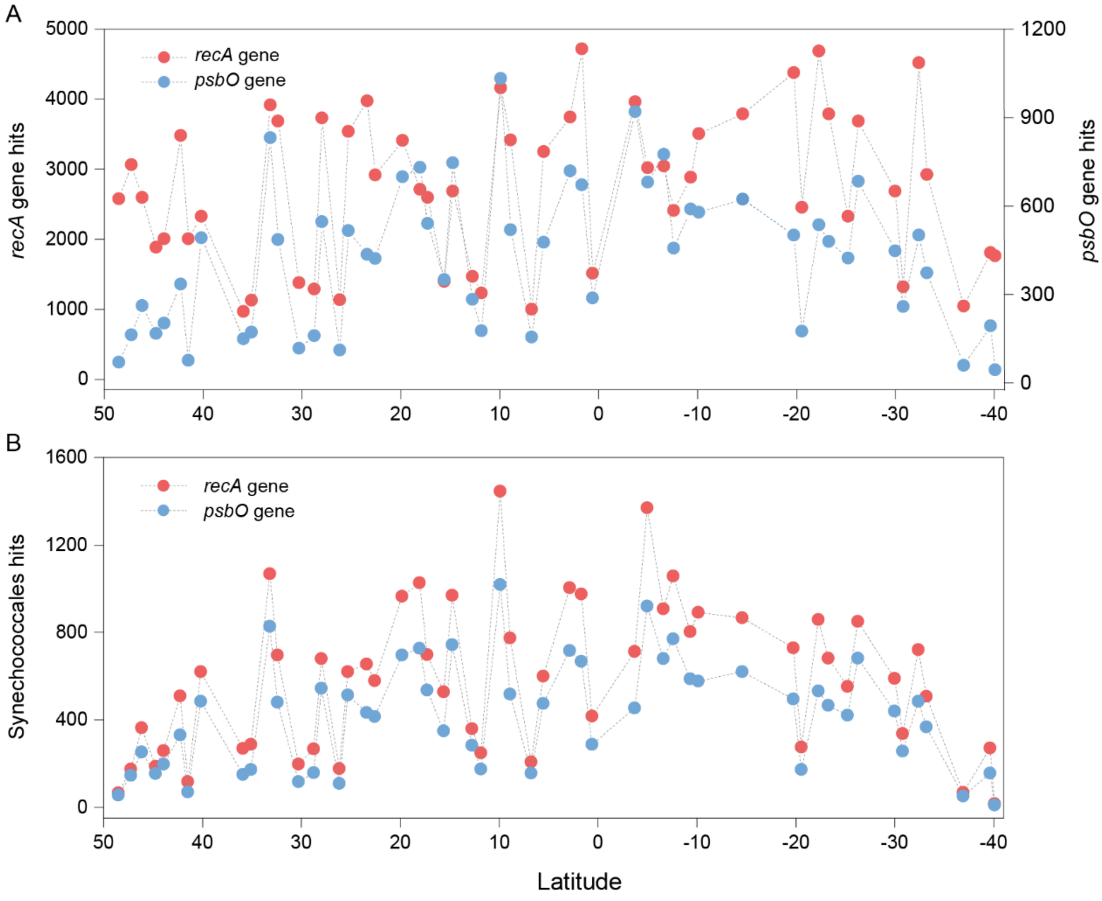

Fig. S3. The number of BLAST hits (**A**) and *Synechococcales* (**B**) based on the single-copy *recA* and *psbO* genes in AMT29 samples.

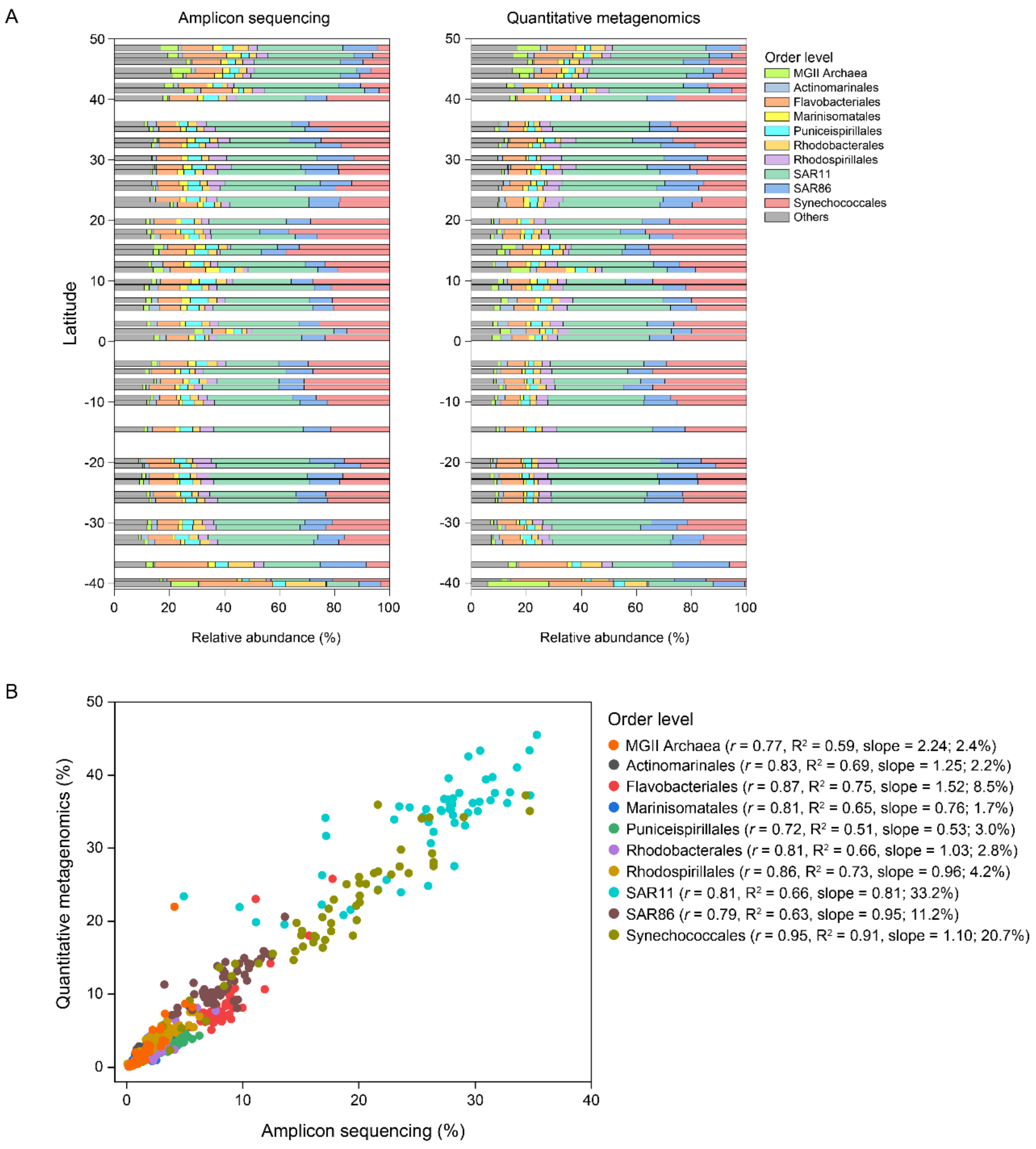

Fig. S4. Comparison of relative abundance of prokaryotes in AMT29 samples. (**A**) Relative abundance of major prokaryotes at the order level derived from amplicon sequencing (left) and quantitative metagenomics (right). The *recA* and *radA* data from quantitative metagenomics were recalculated on relative abundance at the order level. The average relative abundance of each order in metagenomics was indicated in the parentheses right after the regression statistics. (**B**) Pearson correlation between relative abundances estimated by amplicon sequencing and quantitative metagenomics. Correlation coefficients are presented for each order.

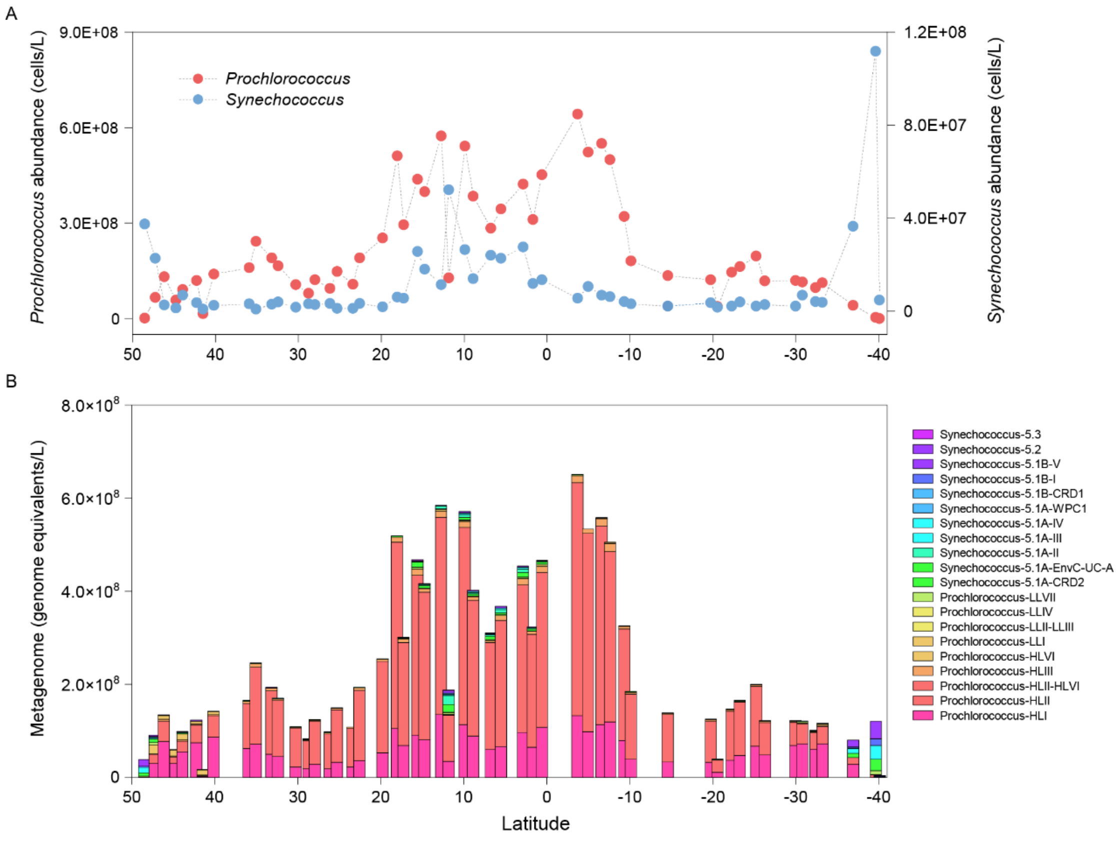

Fig. S5. The absolute abundace of *Prochlorococcus* and *Synechococcus* across the Atlantic Meridional Transect. (**A**) The absolute abundance was estimated using the *recA*-based quantitative metagenomics. (**B**) The ecotype of *Prochlorococcus* and *Synechococcus* from *recA*-based metagenomics was defined using the DIAMOND BLASTx (-k 1, -e 1e-5) against the cyanobacterial clusters of orthologous groups of proteins (CyCOG v6.0 database) [1].

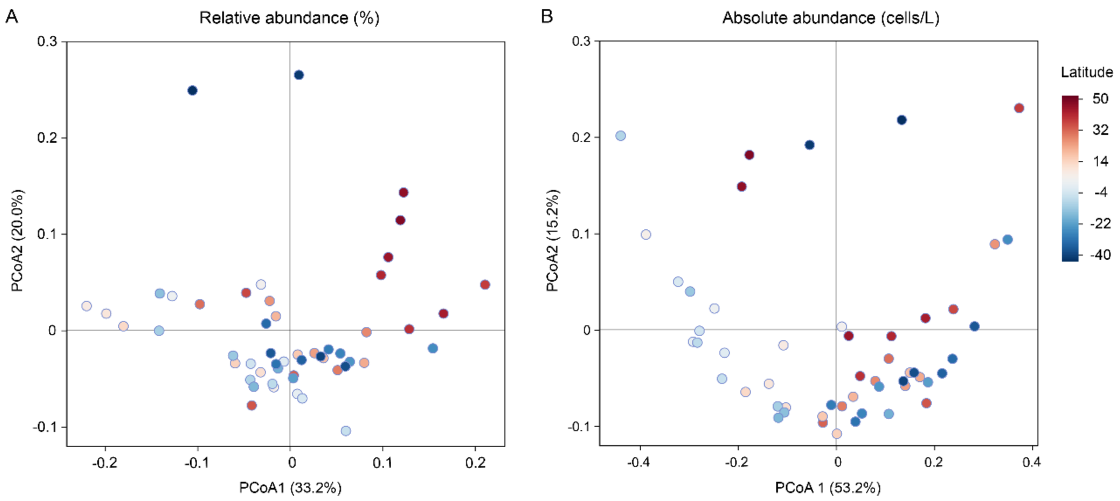

Fig. S6. Order-level bacterial composition of AMT29 samples based upon microbial relative abundance from rRNA gene amplicons (**A**) and absolute abundance from metagenomic hits of *recA* (**B**). Microbial variation between sampling stations was visualized by principal coordinates analysis (PCoA) using Bray–Curtis dissimilarity on the order level matrix. The percentage of variance explained by the two first PCoA dimensions are reported on the axes. Note the higher total variance explained by absolute abundances (68.4%) compared to relative abundances (53.2%).

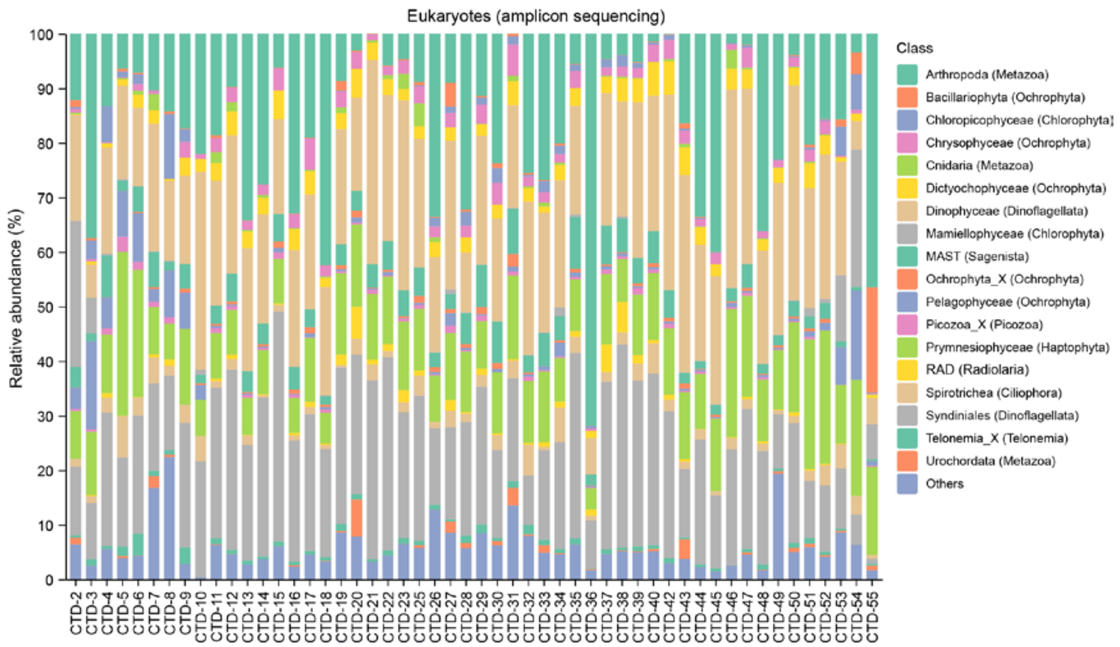

Fig. S7. Taxonomic classification of eukaryotic communities at the class level (including Metazoa sequences) along the AMT29 transect based on 18S rRNA gene amplicon sequencing. Only dominant classes with a relative abundance > 0.05% across all samples were included.

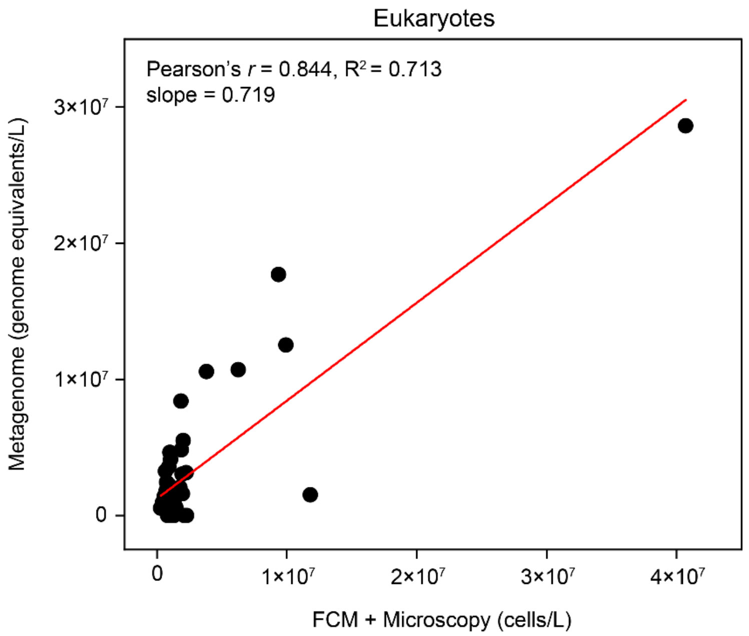

Fig. S8. Relationship between metagenomics-based estimates of eukaryotic absolute abundances and those made by FCM and microscopy. The abundances of diatoms and dinoflagellates and assessed by microscopy, while the cryptophytes, coccolithophores, picoeukaryotes (PEUK), and nanoeukaryotes (NEUK) were assessed by flow cytometry, though note that in practice the PEUK and NEUK constituted > 99% of total abundances overall. For the quantitative metagenomics of eukaryotes, we estimated that the *psbO* gene was present in one copy per haploid genome.

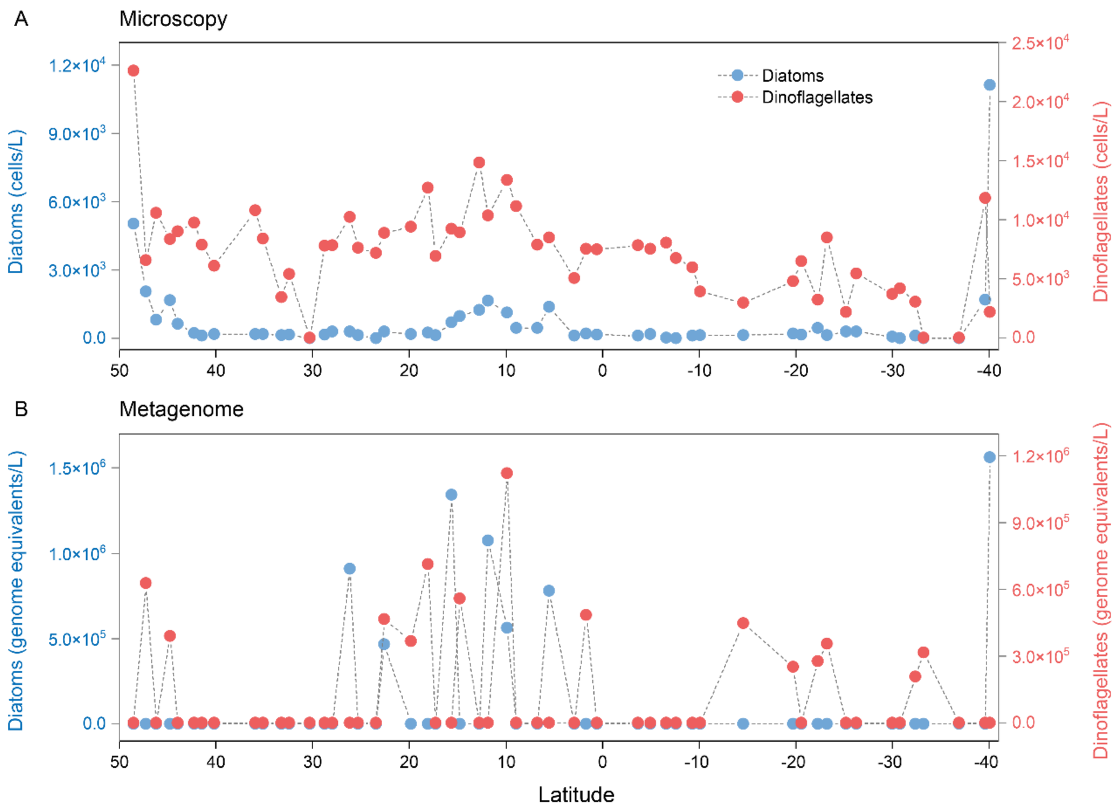

Fig. S9. The absolute abundace of diatoms and dinoflagellates across the Atlantic Meridional Transect using microscopy (**A**) and *recA*-based quantitative metagenomics (**B**). Note that the detection limit is much lower for microscopy, resulting in many zero (undetectable) values in metagenomics. However, when detected by metagenomics, the cell numbers are orders of magnitude larger compared to microscopy.

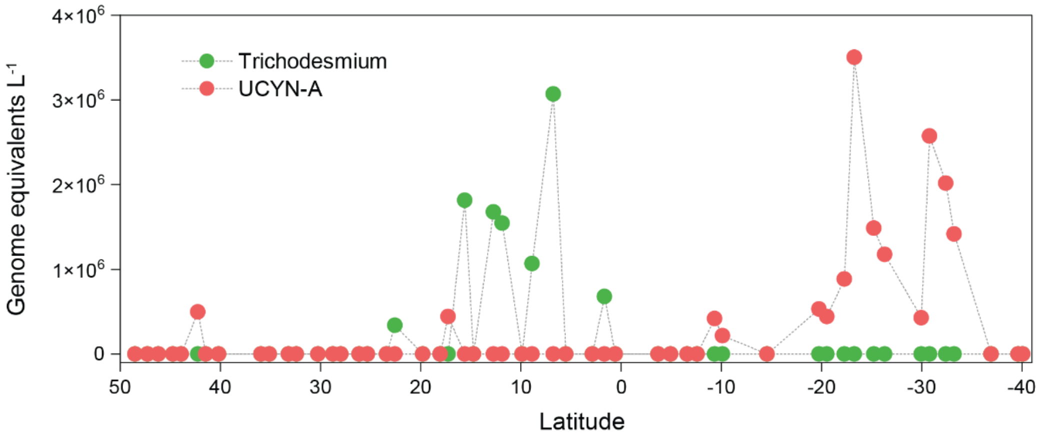

Fig. S10. The absolute abundance of *Trichodesmium* and UCYN-A (*Candidatus* Atelocyanobacterium thalassa) across the Atlantic Meridional Transect using the *recA*-based quantitative metagenomics.

References

1. Berube PM, Biller SJ, Hackl T *et al.* Single cell genomes of prochlorococcus, synechococcus, and sympatric microbes from diverse marine environments. *Scientific data*. 2018;**5**:1-11
